# HIV-1 group O virus identified for the first time in the United States.

**DOI:** 10.3201/eid0203.960307

**Published:** 1996

**Authors:** M A Rayfield, P Sullivan, C I Bandea, L Britvan, R A Otten, C P Pau, D Pieniazek, S Subbarao, P Simon, C A Schable, A C Wright, J Ward, G Schochetman

**Affiliations:** National Center for Infectious Diseases, Centers for Disease Control and Prevention, Atlanta, Georgia, USA.


### HIV-1 Group O Virus Identified for the First Time in the United States

In 1994, seven unusual cases of HIV infection were identified in France (1). These cases occurred in foreign nationals whose specimens had atypical Western blot patterns and weak or no seroreactivity in HIV-1 peptide or recombinant-based enzyme immunoassays (EIA). In previous cases, such isolates had been loosely grouped as phylogenetic outliers, termed Group O strains, highly divergent from the Group M strains that gave rise to the HIV-1 pandemic (2). The 1994 cases were soon recognized as representing the global emergence of genetically distinct viruses known to be circulating in Cameroon and Gabon. This recognition prompted an immediate reassessment of HIV screening in Europe and North America. Subtype B strains of HIV-1 predominate in Europe and North America, and there has been increasing evidence for the entry of non-B strains into these populations through persons who were infected while abroad. However, there was no evidence of indigenous spread of the strains in North America (3,4).

We report here the laboratory findings related to the first documented HIV-1 Group O infection in the United States. The case was identified in April of this year through the CDC sentinel surveillance program for unusual HIV variants and involves a West-Central African woman (residing in the United States), with a 2-year clinical history of lymphadenopathy, thrombocytopenia, and progressive CD4 lymphocyte loss. In February 1995, the patient was tested for HIV-1-specific antibodies and was determined to be seronegative (Abbott HIV-1/2; signal/cutoff value = 0.7) by recombinant-antigen based EIA. The patient's serostatus was reevaluated the following October and November and again in April of 1996 with inconclusive results (Table 1). On these occasions, whole virus lysate-based EIAs yielded negative or weakly positive results (signal/cutoff ratios were 0.68 to 4.20); however, confirmatory Western blot assays remained indeterminate. The November 1995 sample was negative in both Genetic Systems formats; however, a signal/cutoff ratio of 0.89 was noted. The Western blot pattern remained remarkably stable through the year, and the patient's sera remained HIV-1 antigen–negative on each sampling occasion. Attempts by commercial reference laboratories to amplify proviral sequences from the patient's peripheral blood mononuclear cells by standard Group M gag-specific primers for polymerase chain reaction (PCR) or reverse transcriptase (RT)-PCR did not show HIV sequences.

To reconcile the serologic findings, a panel of HIV subtype–specific, peptide-based EIAs was performed at CDC (6) (Table 2). As points of reference, examples of results with sera representing the Group M subtypes A, B, C, D, E, and F are shown. A high degree of cross-reactivity exists among the Group M sera, with the strongest response usually directed against the V3 peptide from the subtype that infected the source patient. The exception to this (Table 2) is seen in the Brazilian subtype F sera, which react strongly with the subtype B peptide but not with the subtype F peptide, which was derived against subtype F isolates from Romania. The distinction between Brazilian and Romanian sera is reflected in the genetic diversity between isolates from the two regions (7). This broad seroreactivity among group M sera is also reflected in their response to group-specific peptides from the gp41. However, the patient's sera were only reactive to the Group O–specific V3 and gp41 peptides (OD = 1.704 and 2.467, respectively). This provided the first direct evidence that this patient was infected with a Group O variant.

The virus CDC7755 was isolated by cocultivating the patient's peripheral blood mononuclear cells with phytohemagglutinin A, which stimulated normal human peripheral blood mononuclear cells. Viral replication was monitored by antigen capture (Coulter SIV Core Antigen Assay, Hialeah, FL) and PCR amplification of the viral protease gene (8). Virus growth was rapid, highly cytolytic, and characterized by punctate syncytial formation throughout the primary culture. All PCR amplifications were performed with hotstart technology and nested primer sets to facilitate direct sequencing. A

Table 1. Results of enzyme immunoassay for antibodies to HIV

| Sample date | Genetic Systems HIV-1 EIA[a] | Genetic Systems HIV-1/2 EIA[a] | OTC HIV-1 EIA[a] | Abbott HIV-1 EIA[a] | Abbott HIV-1/2 EIA[a] |
|---|---|---|---|---|---|
| February 1995 | ND | ND | ND | ND | 0.70 |
| October 1995[a] | 0.51 | 1.35 | ND | ND | ND |
| November 1995[b] | 0.41 | 0.89 | ND | ND | ND |
| April 1996[c] | 4.20 | 3.40 | 0.68 | 3.10 | 1.15 |

[a]HIV-1 WB = indeterminate; detected p17, p24, p31, p50, p66, and equivocal gp41; HIV-1 antigen = negative (0.40).
[b]HIV-1 WB = same as above; HIV-1 antigen = negative (0.34).
[c]HIV-1 WB = same as above; HIV-1 IFA = positive (2.5+).
ND = not done.

Table 2. Typical peptide reactivity patterns.

| Subtype | V3-A | V3-B | V3-C | V3-D | V3-E | V3-F | V3-O | GP41-M | GP41-O |
|---|---|---|---|---|---|---|---|---|---|
| A | 2.742 | 1.144 | 1.628 | 0.065 | 0.083 | 0.724 | 0.055 | 2.683 | 2.627 |
| B | 0.071 | 1.271 | 0.051 | 0.030 | 0.033 | 0.038 | 0.028 | 2.570 | 2.414 |
| C | 2.320 | 0.084 | 2.515 | 0.079 | 1.647 | 1.637 | 0.061 | 2.746 | 2.858 |
| D | 0.210 | 0.079 | 0.056 | 1.203 | 0.048 | 0.051 | 0.040 | 1.380 | 0.279 |
| E | 0.055 | 0.043 | 0.385 | 0.042 | 1.291 | 0.038 | 0.025 | 0.619 | 0.340 |
| F | 0.062 | 0.657 | 0.041 | 0.037 | 0.039 | 0.033 | 0.037 | 2.615 | 2.670 |
| O | 0.089 | 0.088 | 0.089 | 0.188 | 0.088 | 0.081 | 1.704 | 0.070 | 2.457 |

GP41-M and GP41-O refer to peptides derived from the consesus gp41 sequences for Group M and Group O variants.

consensus set of nested primers, which is generic for Group M viruses, was first used to attempt amplification and detection of the viral protease. This set did not detect proviral sequences during direct evaluation of the patient's peripheral blood mononuclear cells; however, a comparably derived consensus primer set for the Group O proteases readily amplified proviral sequences from the patient's cells and cocultures.

For sequence comparison and phylogenetic analysis, proviral sequences spanning the viral protease gene and portions of the p24 and gp120 gene regions were amplified from the patient's peripheral blood mononuclear cells and cocultures.[1] Amplification of a 738-bp fragment spanning the junction of the p24 and p17 genes of the gag region was accomplished through a nested set of generic primers (9). Group O consensus primer sets were derived to amplify the 297-bp viral protease gene and a 381-bp fragment spanning the C2V3 portion of gp120 within the env gene. Sequence identity was demonstrated between repeated patient samplings and successive days in cocultivation for each of the gag, pol, and env elements, thus confirming a common but unique source for the isolate. For phylogenetic analysis, these sequences were further edited to obtain the relevant portions of the protease gene (254 bp), p24 (419 bp), and C2V3 region( 233 bp) of gp120. Phylogenetic analysis was performed by neighbor-joining and maximum-likelihood methods (10,11).

In the phylogenetic analysis of the protease gene, we included the two available Group O prototypes (ANT70 and MVP5180), reference strains for the HIV-1 subtypes representing Group M variants, and HIV-2 strains (Figure 1A). The fidelity of the phylogenetic results was verified by boot strap analysis (100 data sets) and pruning, i.e., the sequential removal of representative strains followed by reanalysis. The segregation of CDC7755 and Group O strains was absolute and is consistent with a divergence independent of the rooting of the Group M clades (Figure 1A). For each gene region, all resulting trees were consistent with the exclusion of the CDC7755 isolate from the Group M family and its strong inclusion with the Group O strains.

To better understand the evolutionary relationship between the patient s strain and other Group O isolates, we analyzed the C2V3 region of gp120 from multiple Group O strains. This gene region exhibits a high degree of divergence and has been sequenced for the largest number of Group O variants (Figure 1B). The chimpanzee isolate SIV-CPZ was used as an outgroup. We took this approach to avoid possible errors introduced through an alignment of very distant HIV-1 and HIV-2 strains. Our findings indicate that strain CDC7755 retains a distant evolutionary relationship to the MVP5180 isolate (Figure 1B, MVP51) yet represents a distinct evolutionary lineage within Group O. The nucleotide sequence divergence among the represented strains is 19% to 26%, values characteristic of Group O viruses and typical of intersubtype rather than intrasubtype genetic distances among the Group M variants. This level of diversity among the Group O members emphasizes the potential that the limited number of “O” variants yet recognized may represent a set of genetic lineages as distinct as the Group M subtypes.

An alignment of the deduced C2V3 amino acid sequence of strain CDC7755 with those of three Cameroonian Group O reference strains is presented in Figure 2; the V3 loop is bound by the two cysteine residues (residues 45 and 83). Unlike Group M viruses, which show greater heterogeneity in the regions flanking the V3 loop, the Group O viruses, including the CDC7755 strain, show substantial divergence within the loop itself. The pattern of potential N-glycosylation sites over the C2V3 region of the CDC7755 strain is consistent with that found

[1]Primers: gag/outer/forward - AGTACATGTTAAAACATGTAGTATGGGC; gag/outer/ reversed - CCTACTCCCTGACAGGCCGT CAGCATTTCTTC; gag/inner/forward - AGTACATGTTAAAACATGTAGTATGGGC; gag/inner/reverse - CCTTAAGCTTTTGT AGAATCTATCTACATA; protease/outer/forward - TTTGCCTCCCTCAAATC; protease/outer/reverse - TTACTGGCACTG GGGCTATGG; protease/inner/ forward - CCTCAAATCCCTCTTTG; protease/inner/reverse - TATAGGGAAGTTTAGTGTACA; env/outer/forward - CACAGAATTTAATGGAACAGGC; env/outer/reverse - TGTGTT ACAATAAAAGAATTCTCC; env/inner/forward - GTTACTTGTACACATGGCAT; and env/inner/reverse - AGAATTCTCCATGACAGTTAAA.

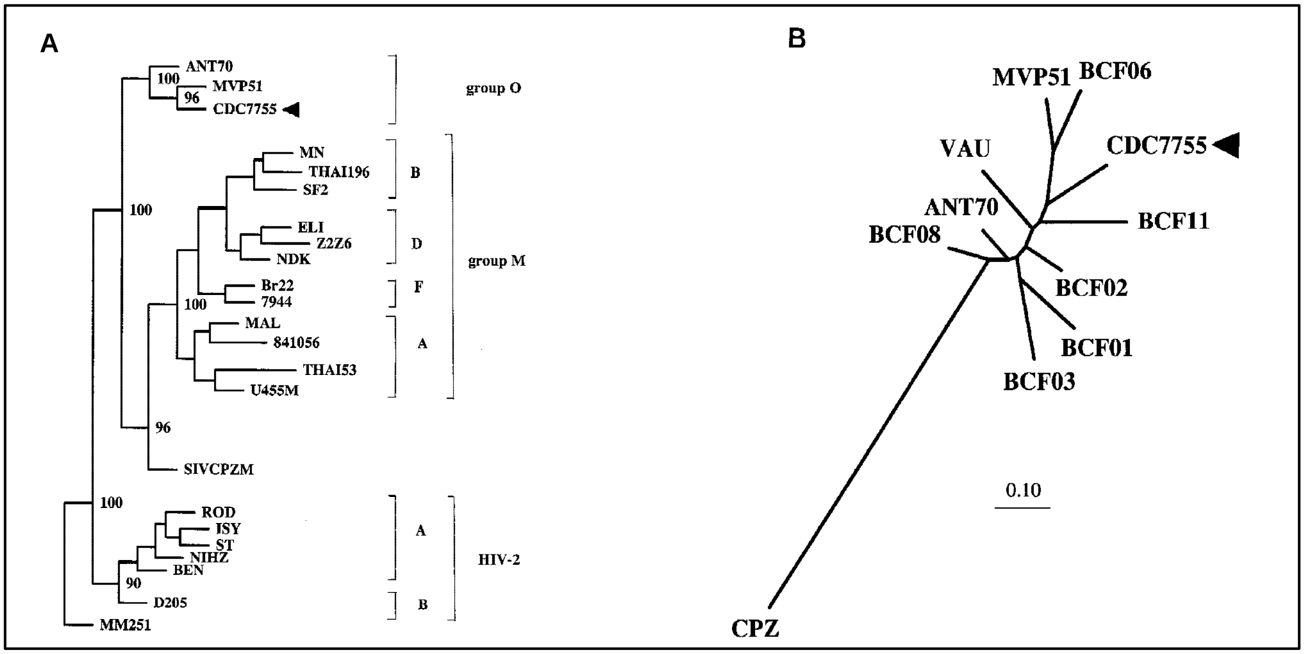

Figure 1. Phylogenetic analysis of the prt and env gene sequences. The patient's isolate is indicated by the arrow. The trees were constructed on the basis of the proviral DNA sequences for the protease gene (A) by the neighbor-joining method in the Phylip 3.5c package (2) and 230 aligned nucleotides from the C2V3 region of the env gene of Group O isolates (B) by using the maximum likelihood method with the fast DNAml program (1). Numbers at the branch nodes within the protease tree indicate bootstrap values. The SIV-MM251 protease gene sequence and the SIV-CPZ env sequence were used as outgroups in trees A and B, respectively. The nucleotide sequence distance among strains can be deduced from the horizontal branch lengths by using the included bar scale. Vertical distances are for clarity only.

among the Group O reference strains. Also of interest is the increased number of amino acid residues in the V3 loop of the CDC7755 strain (37 amino acids)–a feature common to other Group O viruses but two to three residues more than most Group M viruses; however, the significance of these differences is not clear.

To date, nearly all of the Group O infections reported worldwide (fewer than 100), have been detected in persons from West-Central Africa. This is the first Group O case reported in the United States, and the presence of these infections here is believed to be exceedingly low. However, the discovery of any divergent HIV strains should be considered a sentinel event and should prompt the reappraisal of HIV antibody tests used to protect of the blood supply. In the United States guidelines for preventing HIV transmission by blood and blood products include testing for HIV-1 and -2; the refinements necessary to improve the sensitivity of

| | | | | | |
|---|---|---|---|---|---|
| CONS-O | K P T V S T Q L I L | N G T L S K G K I R | I M G K N I S E S G | K N I ? V T L N ? T | I N M T C E R P G ? |
| ANT70 | R - - - - - - - - - | - - - - - - - - - - | M - A - D - L - G - | - - - I - - - - S - | L - - - - - - - Q I |
| MVP5180 | - - - - - - - - - - | - - - - - - R E - - - | - - - - - - - T - - A | - - - I - - - - T P | - - - - - - I - E - I |
| VAU | - - - - - - - - - - | - - - - - - - - N - T | - - - - - - - D - - | E - - L I - - - T N | - T I A - - - - - N |
| CDC7755 | - - - - - - H - - F | - - - I - - - - - - | - - V - - - - S N S | D - - L - - - - S - | - - - - - - V - - - N |
| | | | | | |
| CONS-O | ? ? ? Q E ? R I G P | M A W Y S M G L G R | T ? G ? ? ? ? S R A | A Y C T Y N A T D W | ? K A L ? Q ? A E R |
| ANT70 | D * I - - M - - - - | - - - - - - - - I - G | - A - N * * S - - - | - - - K - - - - - - | G - I - K - T - - - |
| MVP5180 | A E V - D I Y T - - | - R - R - - T - K - | S N N T S P R - - V | - - - - - - - K - V - | E N - - Q - T - I - |
| VAU | Q T I - K I M A - - | - - - - - - - A - S N | - K - D * * * T - - | - - - N - S - - - - | N - - - K N I T - - |
| CDC7755 | N S V - - M - - - - | L - - - - - - - - - | g Y A S * * K - - I | - F - - - - - - E - | K E T - Q G I - - - |
| | | | | | |
| CONS-O | Y L E L V N ? N T E | N V T ? T F ? ? S S | G G D A E V T H L H | . . . . . . . . . . | . . . . . . . . . . |
| ANT70 | - - - - - - * - - G | S I N M - - N H - - | - - - L - - - - - - | . . . . . . . . . . | . . . . . . . . . . |
| MVP5180 | - - N - - - * Q - - | - - - I I - S R T - | - - - - - - S - - - | . . . . . . . . . . | . . . . . . . . . . |
| VAU | - - - - - E Y - Q T | D - - M K - G - H - | - E - - - - - - N F F | . . . . . . . . . . | . . . . . . . . . . |
| CDC7755 | - - - - - - * H - - | - S - I - - K N - T | - - - I - - - R - - | . . . . . . . . . . | . . . . . . . . . . |

Figure 2. Alignment of deduced amino acid sequences of the env C2V3 region of the CDC7755 strain with those of three representative Group O Cameroonian strains (ANT70C, MVP5180 and FR.VAU). The CONS.O represents the consensus amino acid sequence derived from the four strains presented in this alignment. (?) represents positions where a consensus could not be derived. (-) indicates identical amino acids with the CONS.O and (*) indicates gaps (insertion\deletions) that were introduced to align the sequences.

these diagnostic tests for the detection of Group O variants are known. Other criteria for donor deferral (e.g., self-deferral for donors at high risk for HIV infection and temporary exclusion of donors from malaria-endemic regions) that may confer a measure of protection against divergent HIV viruses present in equatorial Africa are in place. The identification of Group O infections underscores the potential for the emergence of other highly divergent HIV strains and highlights the importance of maintaining active worldwide surveillance for these variants.

**M. A. Rayfield,* P. Sullivan,† C. I. Bandea,* L. Britvan,‡ R. A. Otten,* C. P. Pau,* D. Pieniazek,* S. Subbarao,* P. Simon,† C. A. Schable,* A. C. Wright,* J. Ward,† and G. Schochetman***

*National Center for Infectious Diseases; †National Center for HIV, STD and TB Prevention, Centers for Disease Control and Prevention, Atlanta, Georgia, USA; ‡Kaiser Permanente Medical Group, Los Angeles, California, USA

### References

1. Loussert-Ajaka I, Chaix M, Korber B, et al. Variablility of human immundeficiency virus type 1 group O strains isolated from Camerooniian patients living in France. J Virol 1995;69:5640-9.
2. Myers G, Korber B, Wain-Hobson S, Smith R, Pavlakis G. Eds. Human retroviruses and AIDS 1994: a compilation and analysis of nucleic acid and amino acid sequences. Los Alamos, NM: Los Alamos National Laboratory, 1993.
3. Brodine SK, Mascola JR, Weiss PJ, et al. Detection of diverse HIV-1 genetic subtypes in the USA. Lancet 1995;346:1198-9.
4. Schable C, Zekeng L, Pau CP, et al. Sensitivity of United States HIV antibody tests for detection of HIV-1 Group O infections. Lancet 1994;344:1333-4.
5. Identification of HIV-1 Group O Infection — Los Angeles County, California, 1996. MMWR 1996;45:561-5.
6. Pau CP, Hu DJ, Spruill C, et al. Surveillance for human immunodeficiency virus type 1 group O infections in the United States. Transfusion. 1996;36:398-400.
7. Bandea C, Ramos A, Pieniazek D, et al. Epidemiologic and evolutionary Relationships between Romanian and Brazilian HIV-1 subtype F strains. Emerging Infectious Diseases. 1995;1:91-3.
8. Pieniazek D, Janini LM, Ramos A, et al. [HIV-1 patients may harbor viruses of different phylogentic subtypes: implications for the evolution of the HIV/AIDS pandemic. Emerging Infectious Diseases 1995;1:86-8.
9. Gao F, Yue L, Robertson DL, et al. Genetic diversity of human immunodeficiency virus type 2: evidence for distinct sequence subtypes with differences in virus biology. J Virol 1994;68:7433-47.
10. Larsen N, Olsen GJ, Maidak BN, et al. The ribosomal database project. Nucleic Acids Res 1993;21:3021-3.
11. Felsenstein J. PHYLIP-phylogeny inference package (version 3.2). Cladistics 1989;5:164-6.
